# Peripheral Lymphocytes in Primary Liver Cancers: Elevated NK and CD8+ T Cells and Dysregulated Selenium Metabolism

**DOI:** 10.3390/biom14020222

**Published:** 2024-02-14

**Authors:** Cheng Zhou, Zhufeng Lu, Baoye Sun, Yong Yi, Boheng Zhang, Zheng Wang, Shuang-Jian Qiu

**Affiliations:** 1 Key Laboratory of Carcinogenesis and Cancer Invasion (Ministry of Education), Department of Liver Surgery and Transplantation, Liver Cancer Institute, Zhongshan Hospital, Fudan University, Shanghai 200032, China; zhoucheng16@fudan.edu.cn (C.Z.); 21111210106@m.fudan.edu.cn (B.S.);; 2 Department of Anesthesia, Zhongshan Hospital, Fudan University, Shanghai 200032, China; lu.zhufeng@zs-hospital.sh.cn; 3 Department of Hepatic Oncology, Xiamen Clinical Research Center for Cancer Therapy, Zhongshan Hospital, Fudan University (Xiamen Branch), Xiamen 361015, China; 4 Center for Evidence-Based Medicine, Shanghai Medical School, Fudan University, Shanghai 200032, China

**Keywords:** primary liver cancer, peripheral blood lymphocytes, SELENBP1, SEPP1

## Abstract

Peripheral blood lymphocytes (PBLs), which play a pivotal role in orchestrating the immune system, garner minimal attention in hepatocellular carcinoma (HCC) and intrahepatic cholangiocarcinoma (ICC). The impact of primary liver cancers on PBLs remains unexplored. In this study, flow cytometry facilitated the quantification of cell populations, while transcriptome of PBLs was executed utilizing 10× single-cell sequencing technology. Additionally, pertinent cases were curated from the GEO database. Subsequent bioinformatics and statistical analyses were conducted utilizing R (4.2.1) software. Elevated counts of NK cells and CD8+ T cells were observed in both ICC and HCC when compared to benign liver disease (BLD). In the multivariate Cox model, NK cells and CD8+ T cells emerged as independent risk factors for recurrence-free survival. Single-cell sequencing of PBLs uncovered the downregulation of TGFβ signaling in tumor-derived CD8+ T cells. Pathway enrichment analysis, based on differential expression profiling, highlighted aberrations in selenium metabolism. Proteomic analysis of preoperative and postoperative peripheral blood samples from patients undergoing tumor resection revealed a significant upregulation of SELENBP1 and a significant downregulation of SEPP1. Primary liver cancer has a definite impact on PBLs, manifested by alterations in cellular quantities and selenoprotein metabolism.

## 1. Introduction

Primary liver cancer (PLC), which is the third leading cause of cancer mortality, is classified into hepatocellular carcinoma (HCC), intrahepatic cholangiocarcinoma (ICC), and combined hepatocellular-cholangiocarcinoma by pathology [1]. The former two types account for 99% of PLC, while combined hepatocellular-cholangiocarcinoma is generally considered to be a combination of ICC and HCC [2].

It is proposed that the neoplasm should be seen as a systemic disease despite usually being found in particular organs [3]. Despite the roles of immunity and angiogenesis being recognized in tumor development [4], PBLs are rarely studied in solid tumors. Most researches focus on the immune microenvironment within the tumor or peritumoral tissues, with limited understanding of how tumors affect immune cells in the peripheral blood.

Systemic therapies, particularly immunotherapies, occupy an important position in the comprehensive treatment of PLC since many patients are ineligible for surgery at diagnosis, and the recurrence rate following surgery is high [5]. Immunotherapies have received much attention in the last decade, but have a limited efficacy in PLC [6]. Therefore, it is essential to gain a deeper understanding of the immune characteristics in liver cancer. PBLs, as a critical component of the immune system, have not been well studied, and there is a lack of research on this topic. One major obstacle in tumor immunotherapy is the functional exhaustion of T cells, which hinders the effectiveness of immune checkpoint inhibitors [7]. While efforts have been made to reverse or delay T cell exhaustion near tumor sites, it has been reported that T cells within tumors may not respond well to immune checkpoint therapy [8]. Some researchers believe that the state of exhausted T cells is irreversible, leading to an inability to sustain immune responses to immune checkpoint inhibitors [9]. To establish sustained anti-tumor immunity, a continuous influx of effector T cells from the peripheral blood into the tumor is crucial.

In recent years, single-cell sequencing technology has made significant advancements and has emerged as a powerful tool for studying the immune microenvironment. This technology allows for the detection of individual cell transcriptome information, enabling researchers to explore intercellular interactions and regulatory networks at the single-cell level. This approach provides a more comprehensive and novel understanding of the biological characteristics of PLC. Previous studies using single-cell sequencing in liver cancer have revealed the enrichment of exhausted CD8+ T cells and regulatory T cells in the tumor, the expansion of TCR clonality [10], the potential migration of LAMP3+ dendritic cells from tumors to lymph nodes [11], and the regulation of various lymphocyte subtypes. Additionally, similarities have been observed between endothelial cells, macrophages, and embryonic liver development processes within the liver cancer ecosystem [12]. CD8+ T cells in recurrent liver cancer exhibit lower cytotoxicity compared to those in primary liver cancer and are characterized by a high expression of KLRB1 [13,14]. However, these studies have not focused on PBLs from liver cancers.

In our study, we conducted flow cytometry analysis to determine the cell counts of peripheral blood lymphocytes (PBLs) in benign liver disease (BLD), HCC, and ICC. Additionally, we employed single-cell sequencing to investigate the features of cell communication among PBLs from ICC and HCC patients. Through these analyses, we aim to contribute to a better understanding of the role of PBLs in liver cancers. 

## 2. Materials and Methods

### 2.1. Patients and Follow-Up

This study was approved by the Institutional Ethics Committee of Zhongshan Hospital, Fudan University (B2019-216R) and was conducted in accordance with the Helsinki Declaration ((World Medical Association Declaration of Helsinki, 2013). Retrospective analyses were carried out based on the medical records of patients from January 2013 to December 2019. Patients with malignant tumors were followed up in the clinic once every month in the first postoperative year and once every 3–4 months thereafter. The patient selection is detailed in Supplementary Figure S1. The median follow-up period was 38.6 months (ranging from 1.0 to 73.6 months). All patients underwent surgical resection exclusively and did not receive any other anti-tumor treatment prior to tumor recurrence.

### 2.2. Cell Counting of Peripheral Blood Lymphocyte

In this study, the cell percentages of the peripheral blood lymphocytes (PBLs) were determined by the departments of laboratory medicine at Zhongshan Hospital. The specific cell populations analyzed included CD8+ T cells, CD4+ T cells, NK cells, and B cells. All cell counts for the same patient were derived from a single assessment using a BD (New Jersey, USA) FACSCanto II flow cytometry machine. CD8+ T cells were defined as cells that were double-positive for CD3 and CD8. CD4+ T cells were defined as cells that were double positive for CD3 and CD4. NK cells were defined as cells that were positive for CD56 or CD16 and negative for CD3. B cells were defined based on the expression of CD19.

### 2.3. Single-Cell Sequencing and iTRAQ

In this study, the sequencing procedures were conducted by Genergy Biotechnology, Ltd. (Shanghai, China). The specific sequencing platform was the Novaseq 6000 system from Illumina. Before sequencing, single-cell separation and library construction were carried out using the 10× Chromium 5’ Library & GelBead and 10× Genomics Chromium technologies (Pleasanton, CA, USA). To ensure the quality of the sequencing data, a Bioanalyzer Agilent 2100 instrument from Agilent Technologies (Santa Clara, CA, USA) was employed for quality control. This study included a total of four patients with ICC and two patients with HCC. Additionally, four healthy controls from the GSE198616 dataset and two HCC patients from the GSE140228 dataset were included in this study. Blood protein samples were achieved using the SDT fragmentation method. Each sample was taken with 100 μg peptide segments and labeled according to the instructions of the iTRAQ labeling kit from the AB SCIEX company (Toronto, Canada). The following iTRAQ was conducted by Shanghai Genechem Co., Ltd. (Shanghai, China).

### 2.4. Bioinformatics Analyses

In this study, the raw sequencing data of the cells were processed up to the FASTQ format, and the human genome reference hg38 was used for alignment. Cellranger V3.1 was employed for quality control and data filtration. Doublet prediction was performed using the scDblFinder V1.9.1 [15]. Principal component analysis was performed for dimension reduction, followed by clustering using the Louvain algorithm. Harmony was followed for removing batch effects across samples [16]. KEGG and GSVA analyses were conducted using the clusterProfiler package [17]. Cell communication analysis was conducted using the Cellchat package. Genes with *p* < 0.05, fold change > 2, and PCT > 0.1 were considered relevant. The R-software packages used in this study are listed in Supplementary Table S1. The methods of bioinformatics analyses are described in detail in our previous study [18].

### 2.5. Statistics

The Mann-Whitney U test and t-test were used to compare continuous variables. The x-tile V3.6 software was utilized to determine the optimal cut-off values. Survival analysis was performed using the Kaplan-Meier method. The log-rank test was used to compare the survival curves between different groups. Factors that showed a *p*-value less than 0.10 in univariate analyses were included in the multivariate Cox proportional hazards model. A 2-tailed *p*-value less than 0.05 was considered statistically significant.

## 3. Results

### 3.1. The Counts of Peripheral NK Cells and CD8+ T Cells Elevated in Primary Liver Cancers

Using flow cytometry, we calculated the percentages of PBLs by flow cytometry from 53 ICC patients, 336 HCC patients, and 58 BLD patients (including 45 cases of hepatic hemangioma and 13 cases of liver cysts). Additionally, cell counts were also measured in 346 patients (Supplementary Table S2). Among the entire cohort, two individuals had hepatitis A, five with hepatitis C, and four with hepatitis D. Within the population of HCC patients, 283 out of 336 had hepatitis B. In contrast, the proportions of ICC and BLD patients with hepatitis B were 6 out of 53 and 6 out of 58, respectively.

**Figure 1:**
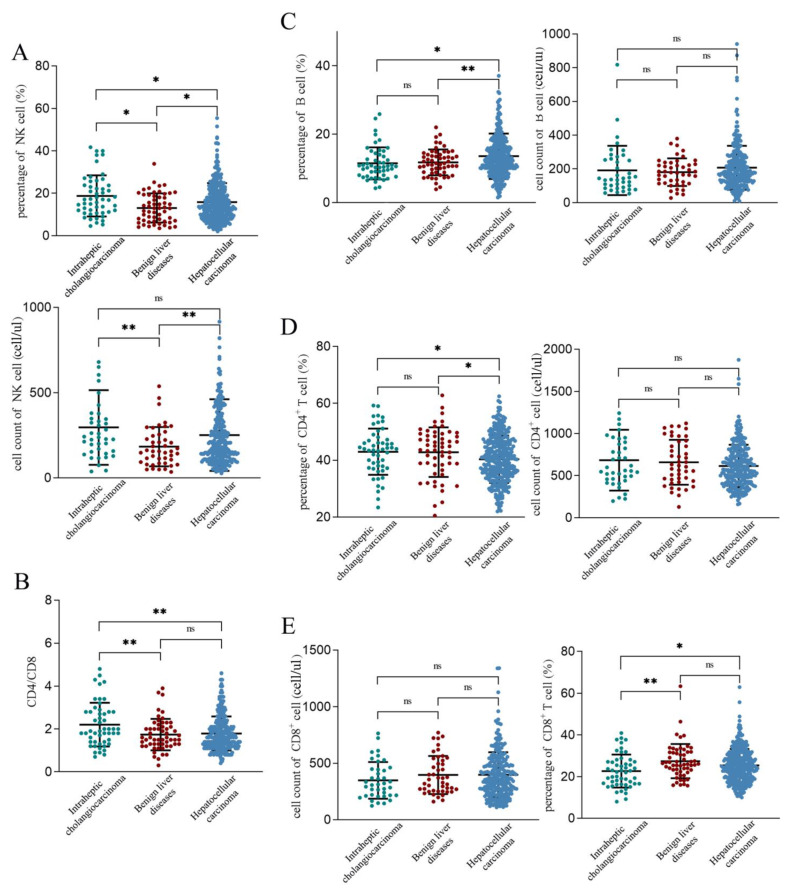
Percentages and cell counts of NK cells (**A**), B cells (**C**), CD4+ T cells (**D**) and CD8+ T cells (**E**). The CD4+/CD8+ T cell ratio of intrahepatic cholangiocarcinoma was also higher than that in hepatocellular carcinoma and benign liver diseases (**B**). * *p* < 0.05, ** *p* < 0.01, ns, no significance.

The percentages (ICC: HCC: BLD = 18.7% vs. 15.8% vs. 13.1%) and cell counts (ICC: HCC: BLD = 296 vs. 251 vs. 183 cells/μL) of NK cells were significantly higher in both the ICC and HCC groups compared to those in the BLD group (Figure 1A). The percentage of NK cells in the ICC group was higher than that in the HCC group (*p* = 0.032), although the cell counts did not exhibit a significant difference (*p* = 0.220, Figure 1A). The CD4+/CD8+ T cell ratio of the ICC patients was also higher than that in the HCC (*p* = 0.001) and BLD (*p* = 0.007) patients, while there was no significant difference observed between the HCC and BLD groups (ICC: HCC: BLD = 2.20 vs. 1.79 vs. 1.73, Figure 1B). Additionally, significant differences were observed in the percentages of B cells (HCC: BLD= 13.6% vs. 11.8%, *p* = 0.047) and CD4+ T cells (HCC: BLD= 40.4% vs. 42.8%, *p* = 0.042) between the HCC and BLD groups (Figure 1C,D). The percentage of CD8+ T cells in the ICC group was significantly lower than that in the BLD group (ICC: BLD = 22.7% vs. 27.4%, *p* = 0.003), and there were also significant differences in the percentages of CD4+ and CD8+ T cells between the ICC and HCC patients (Figure 1E).

Given the notably higher prevalence of hepatitis B among HCC patients compared to ICC and BLD patients (all *p* < 0.050), we proceeded to conduct an in-depth analysis of PBLs based on hepatitis B. The findings indicated that HCC patients with a history of hepatitis B infection (HbsAg (+)) exhibit a significantly elevated peripheral blood NK cell count compared to those without the infection (HBsAg (+): HBsAg (−) = 259 vs. 203 cells/μL, *p* = 0.030). Conversely, no significant differences were observed for the other cell types. Furthermore, when performing differential analyses on PBL in patients without hepatitis infection, the results revealed that the NK cell counts in ICC patients remain significantly higher than those in BLD patients (ICC: BLD = 308 vs. 178 cells/μL, *p* = 0.005). In contrast, no significant differences were identified between HCC and BLD patients (HCC: BLD = 213 vs. 178 cells/μL, *p* = 0.144). These outcomes suggest a potential association between elevated NK cells and hepatitis B virus infection.

### 3.2. Peripheral CD8+ T Cell and NK Cells Are Independent Risk Factors for Prognosis

**Figure 2:**
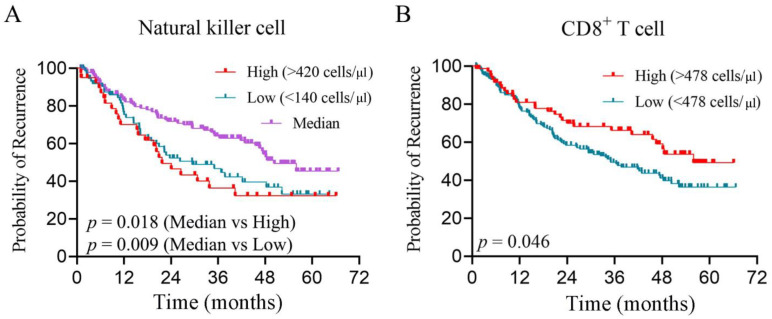
(**A**) Kaplan–Meier survival curves showing that the recurrence-free survival time of patients with low (<140 cells/μL) or high (>420 cells/μL) NK cell levels was shorter than that of those with median NK cell levels in hepatocellular carcinoma. (**B**) The recurrence-free survival time of patients with high (>478 cells/μL) CD8+ T cell levels was longer than that in patient with low CD8+ T cell levels in hepatocellular carcinoma.

After excluding a small number of lost-to-follow-up patients (Supplementary Figure S1 and Supplementary Table S3), we performed survival analysis to determine any potential association between lymphocytes and prognosis in 257 HCC patients with cell counts. The median follow-up time was 36.1 months, but most patients did not reach the follow-up endpoint of overall survival and 46.6% of patients either died or experienced tumor recurrence during the follow-up period. Hence, recurrence-free survival (RFS) was chosen as the focus of this study. X-tile was applied to identify the best cut-off values for immunocytes. Prognostic factors that were associated with the RFS of HCC included CD8+ T cell count, NK cell count, B cell count, and maximal tumor size (Table 1). Notably, we found that both high (>420 cells/μL, *p* = 0.018) or low (<140 cells/μL, *p* = 0.009) NK cells in HCC indicated a poor prognosis (Figure 2A). A high CD8+ T cell count (>478 cells/μL, *p* = 0.046) was an indicator of longer RFS (Figure 2B). In the multivariate Cox modeling, NK cell counts and CD8+ T cells were identified as independent risk factors for the RFS of HCC (Table 1).

In summary, patients with PLC exhibited significant differences in the cell percentage and counts. NK cells and CD8+ T cells not only differ in cell numbers and percentages between PLC and BLD but are also independent risk factors for RFS in HCC.

### 3.3. Single-Cell Sequencing Reveals Transcriptomic Features of Peripheral CD8+ T Cells and NK Cells

To further explore the PBLs variances between PLC patients and healthy individuals, we conducted single-cell sequencing on PBLs obtained from four ICC patients and two HCC patients. As a healthy control (HC), we included four cases from the GSE198616 dataset in the GEO. Additionally, two HCC cases from the GSE140228 dataset were included in the HCC group. After applying quality control measures, we obtained a total of 17,227 cells from HCC patients, 17,682 cells from ICC patients, and 19,030 cells from the HC for further analysis.

**Figure 3:**
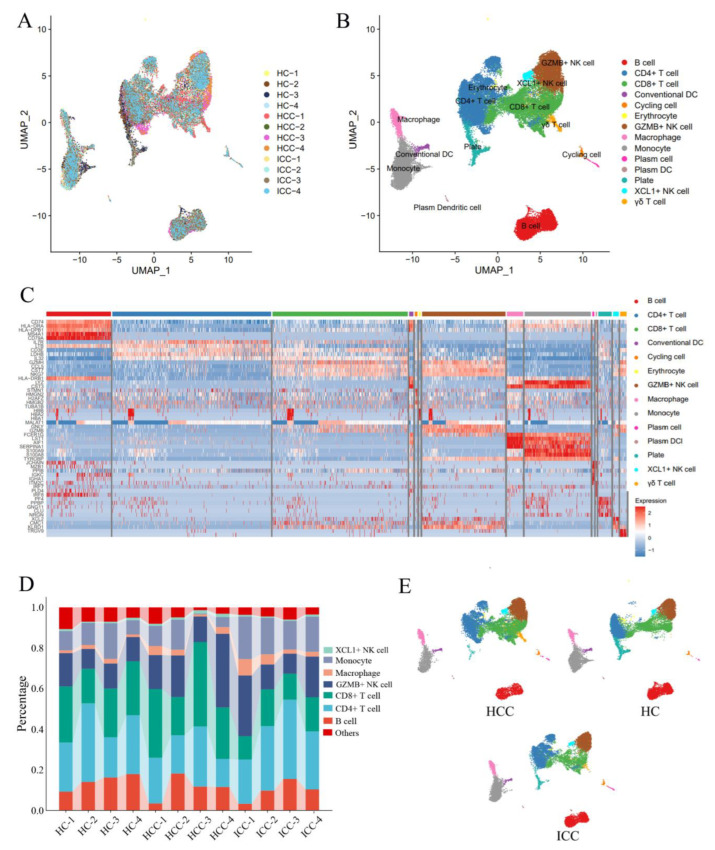
(**A**) A total of 53,939 cells from 12 samples were uniformly distributed in the umap plot, indicating branch effect removal. (**B**) All cells were categorized into GZMB+ NK cells, CD4+ T cells, CD8+ T cells, B cells, XCL1+ NK cells, and so forth. (**C**) The differential genes of all of the cell types. (**D**) The percentages of main cell types among the twelve samples. (**E**) The cell distributions of Peripheral blood lymphocytes were consistent with each other in HCC, ICC, and healthy controls. HCC, hepatocellular carcinoma; ICC, intrahepatic cholangiocarcinoma; HC, healthy control.

After batch effect removal, the PBLs from the different samples showed a uniform distribution (Figure 3A). A total of 53,939 cells were then divided into 32 clusters through dimensionality reduction clustering (Supplementary Figure S2A). These cell clusters were categorized into NK cells, CD4+ T cells, CD8+ T cells, B cells, macrophages, monocytes, and others based on the expression of feature genes (Supplementary Figure S2B) and differential gene expression between subclusters (Figure 3C). Importantly, there was no significant difference in the percentage of cell types between the samples (Figure 3D). Furthermore, no tumor-specific cell subpopulations were observed among these clusters, indicating that the tumors did not induce the formation of any new subtypes of immune cells (Figure 3E). It is possible that changes in peripheral immunocytes are reflected in the alteration in the percentages of cell subtypes.

**Figure 4:**
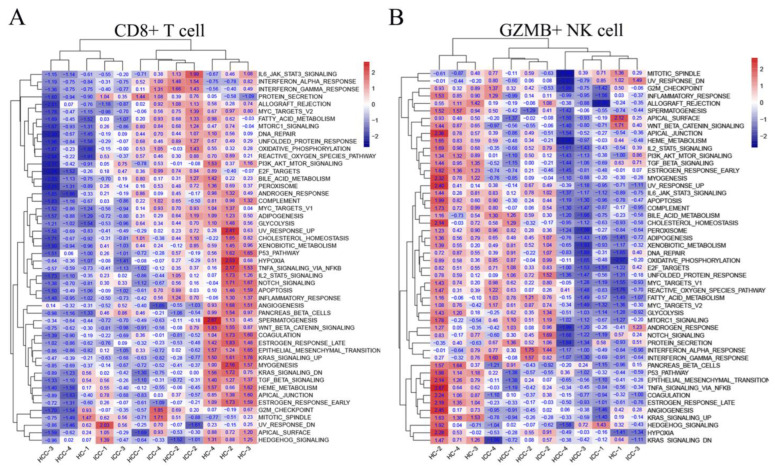
Heatmap showing the results of GSVA signaling pathway analysis based on hallmark gene sets from MSigDB in CD8+ T cells (**A**) and NK cells (**B**). The transcriptomic characteristics of CD8+ T cells and NK cells in liver cancer patients undergo a substantial alteration.

GSVA signaling pathway analysis was conducted to compare the CD8+ T cells and GZMB+ NK cells using hallmark gene sets from MSigDB [19]. The findings revealed the significant downregulation of the APICAL surface pathway, Wnt beta catenin signaling, and TGFβ signaling pathway in HCC compared to the HC. Similarly, in ICC, the Wnt beta catenin signaling, KRASsignaling, and TGFβ signaling pathway were downregulated compared to the HC in both CD8+ T cells (Figure 4A) and GZMB+ NK cells (Figure 4B). When compared to the HC, PBLs from both HCC and ICC displayed distinct characteristics. The downregulation of the TGFβ signaling pathway was observed in both HCC and ICC. Additionally, a further comparison between HCC and ICC revealed that CD8+ T cells and GZMB+ NK cells from ICC exhibited higher TGFβ signaling compared to those from HCC.

### 3.4. TGFβ Signal Decreased in Peripheral CD8+ T Cells from PLC

**Figure 5:**
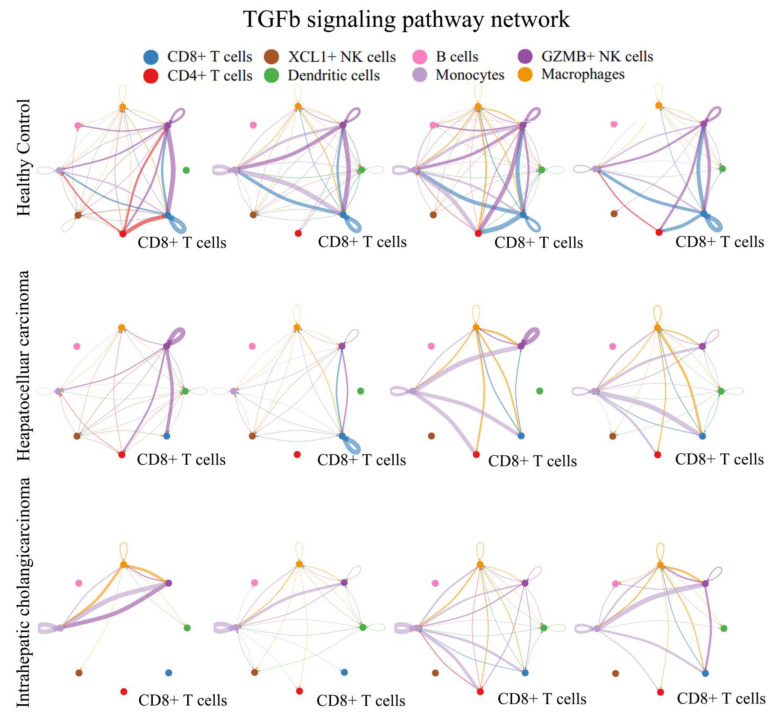
Communication patterns of TGFβ among predominant immune cells were explored using Cellchat. The thicker the line, the stronger the intercellular communication. In healthy individuals, CD8+ T cells assume a prominent role in TGFβ signaling, particularly as senders of the signaling cascade. However, in ICC and HCC, the interaction of CD8+ T cells as signal senders with other immune cells markedly diminishes.

Based on the Cellchat analysis, the communication patterns of PBLs were explored (Supplementary Figure S3). It is noteworthy that CD8+ T cells play a pivotal role in TGFβ signaling in healthy individuals, serving as both primary recipients and senders (Figure 5). However, this pattern is not observed in ICC and HCC. Monocytes and dendritic cells were identified as the main secretory cells of TGFβ in all samples, while CD8+ T cells did not exhibit the significant secretion of TGFβ in ICC or HCC, except for HCC-3, which showed autocrine cell behavior. Based on these findings, the decreased secretion of TGFβ in CD8+ T cells could be a distinguishing feature of PBLs in HCC. CD8+ T cells assume distinct roles in the TGF pathway between healthy individuals and PLC patients.

### 3.5. Differential Genes Enriched in Selenium Metabolism

**Figure 6:**
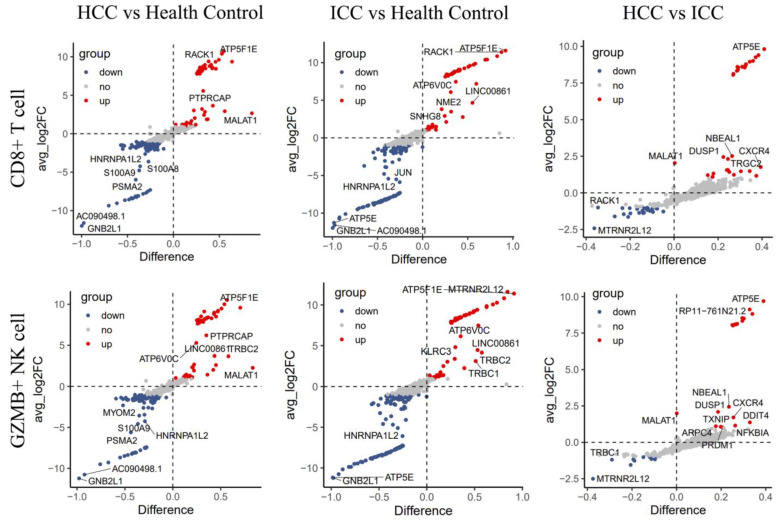
Differential gene analysis was performed on CD8+ T cells and GZMB+ NK cells and genes with *p* < 0.05, fold change > 2, and PCT > 0.1 were considered relevant. HCC, hepatocellular carcinoma; ICC, intrahepatic cholangiocarcinoma.

In order to further analyze the differences in PBLs between PLC and the HC, differential analysis was performed on the CD8+ T cells and GZMB+ NK cells. In the CD8+ T cells, there were 91 upregulated genes and 75 downregulated genes in HCC compared to the HC, and 107 upregulated genes and 105 downregulated genes in ICC compared to HC (Figure 6). In the GZMB+ NK cells, there were 83 upregulated genes and 71 downregulated genes in HCC compared to the HC, and 97 upregulated genes and 104 downregulated genes in ICC compared to the HC (Figure 6). In the differential analysis between ICC and HCC, there were 59 differential genes in the CD8+ T cells and 53 differential genes in the GZMB+ NK cells.

**Figure 7:**
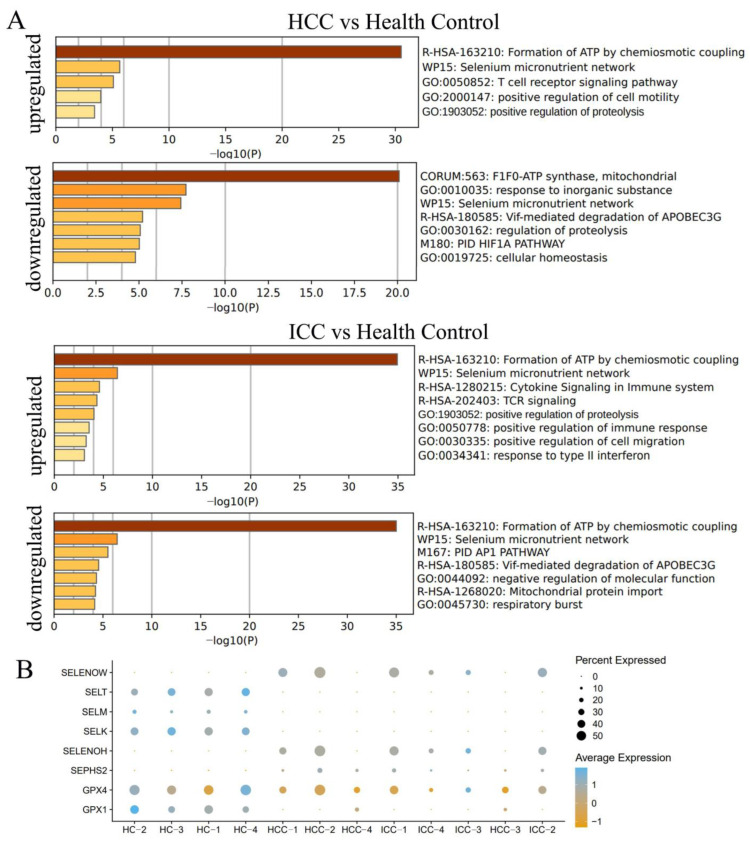
(**A**) Pathway signal enrichment analysis of the upregulated or downregulated genes in CD8+ T cells was conducted using Metascape. The results showed that these differential genes were enriched in ATP formation and selenium metabolism pathways. (**B**) The expression of several selenoproteins in CD8+ T cells varied between primary liver cancers and the healthy control. HCC, hepatocellular carcinoma; ICC, intrahepatic cholangiocarcinoma; HC: healthy control.

**Figure 8:**
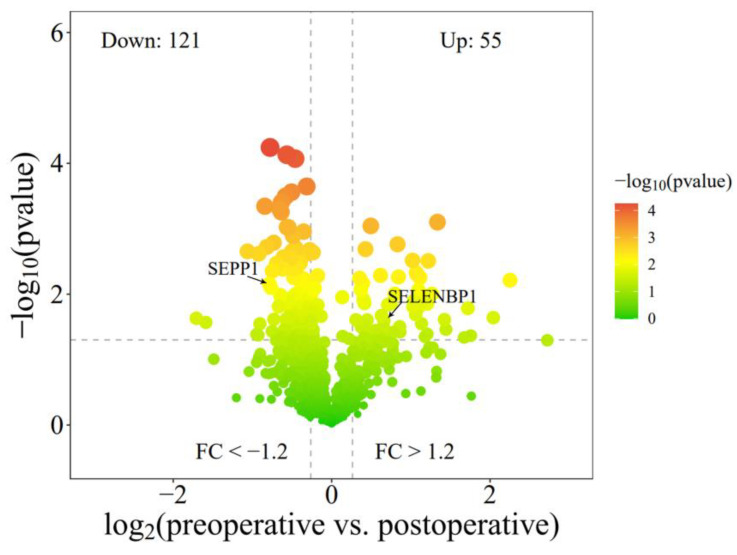
Proteomic analysis results indicate significant decreased SEPP1 and increased SELENBP1 in PBLs after the resection of tumor nodules.

Pathway signal enrichment analysis of the differential genes was conducted using Metascape. The results showed that in both HCC and ICC, whether the genes were up-regulated or downregulated, they were significantly enriched in ATP formation and selenium metabolism pathways (Figure 7A). Further analysis revealed significant differences in the expression of multiple selenoproteins in CD8+ T cells (Figure 7B). Proteomic analysis was conducted on preoperative and postoperative PBLs from patients undergoing tumor resection. The results indicated a significant upregulation of SELENBP1 (*p* = 0.046) and a significant downregulation of SEPP1 (*p* = 0.003) after surgery (Figure 8 and Supplementary Table S4). These findings suggest that there are significant changes in cell metabolism-related functions in peripheral CD8+ T cells and GZMB+ NK cells. The type of selenoprotein in PBL undergoes a notable alteration in cancer patients.

## 4. Discussion

In the past few decades, there have been significant advancements in our understanding of neoplasms, leading to a greater recognition of the role of the immune system in tumor development and progression. However, PBLs, a critical component of the human immune system, have often been overlooked in the study of solid tumors. Despite the recognition that tumors are systemic diseases, and the emergence of studies on circulating tumor DNA [20] and circulating tumor cells [21], the investigation of the relationship between PBLs and PLC remains largely unexplored. In this study, we aimed to address this gap by comparing PBL counts and percentages between patients with PLC and those with BLD. Additionally, we utilized single-cell sequencing to uncover differences in the transcriptome profiles of PBLs between tumor patients and healthy controls. Through these efforts, we hope to shed light on the potential significance of PBLs in PLC and contribute to a deeper understanding of tumor-immune interactions.

The results of the large-scale flow cytometry analysis demonstrated significant differences in the percentage and count of PBLs between patients with PLC and those with BLD. Furthermore, we conducted an analysis to investigate the correlation between lymphocyte counts and the RFS of HCC, with a specific focus on CD8+ T cells and NK cells. Both of these cell types were found to be correlated with the prognosis of HCC. Interestingly, it was observed that both low and high NK cell counts were indicative of a poor prognosis, suggesting that NK cells may exist in at least three different states or undergo transitions through two stages during the development of HCC. Additionally, single-cell sequencing analysis revealed that NK cells derived from PBLs were divided into three states in the trajectory analysis [22], which aligns with our findings. It is important to note that the study of PBLs and their relationship with PLC is still in its early stages, and further research is required to fully understand the underlying connections.

Previous studies have identified several transcriptional features in the PBLs of HCC that are associated with prognosis and diagnostic markers. For example, CLDN18 on PBLs has been established as a prognostic predictor for HCC [23]. The presence of LinlowCD33+HLA-DR- myeloid-derived suppressor cells in the PBLs of HCC patients was found to be increased compared to in healthy individuals [24]. Additionally, the combination of AHNAK and STAP1 methylation in PBLs has shown potential as a diagnostic marker for HBV-related hepatopathy [25], and LGF2 methylation levels were found to be altered in peripheral blood cells [26]. These studies indicate that PBLs are influenced or regulated by tumors, but further research is needed to attribute these changes more precisely. Single-cell sequencing has emerged as a powerful tool for immunological research, allowing for cell classification and theanalysis of intercellular communication. In our study, we performed single-cell sequencing on PBLs from PLC patients and combined it with data from healthy controls obtained from public databases. Through functional pathway analysis, we observed characteristic transcriptome changes in HCC and ICC patients compared to the healthy controls. Both HCC and ICC exhibited the significant downregulation of the TGFβ pathway. Further analysis of the cell communication revealed that changes in the TGFβ signaling pathway in primary liver cancer were primarily due to the downregulation of TGFβ expression in CD8+ T cells. Numerous studies have demonstrated that tumors possess strong regulatory capabilities within the immune microenvironment both within and surrounding the tumor [27], and this is also true in liver cancer [28]. Our findings suggest that tumors may have the ability to remotely regulate peripheral blood lymphocytes, highlighting the complex interactions between tumors and the immune system.

In this study, another noteworthy discovery pertains to the impact on ATP and selenium metabolism pathways. Selenium, as a trace element, plays a complex regulatory role in various immune cells. For example, it serves as an essential regulator of T cell responses and exhibits potential as a therapeutic target in Crohn’s disease [29]. Experiments conducted on mice lacking selenoproteins in their T cells have demonstrated a reduction in the populations of mature and functional T cells in lymphoid tissues, along with impaired antibody responses that rely on T cells [30]. Within the realm of cancer immunity, selenium enhances the lytic activity and cytotoxicity of NK cells [31] and CD8+ T cells [32]. In this study, both upregulated and downregulated genes were found to be enriched in the selenium pathway, indicating intricate changes in selenium levels within PBLs in liver cancers.

The results of this study are limited due to restrictions in funding and the sample size. Achieving more refined classifications and conducting in-depth functional analysis in CD8+ T cells would indeed be valuable for the development of a deeper understanding of the immunological features and pathways involved in liver cancer. Refining cell classifications can provide more detailed insights into the heterogeneity of immune cell populations, allowing for a better understanding of their specific roles and functions in liver cancer. Additionally, conducting more comprehensive functional analyses, including pathway validation and exploring the functional implications of different cell subsets, can help uncover the underlying mechanisms and potential therapeutic targets.

## 5. Conclusions

In summary, our findings reveal elevated counts of peripheral NK cells and CD8+ T cells in HCC and ICC compared to BLD, serving as indicators of RFS for HCC patients. The transcriptional changes in the CD8+ T cell result in the downregulation of the TGFβ pathway in HCC and ICC. Furthermore, abnormalities in selenium protein metabolism are observed in CD8+ T cells and NK cells, manifested by the upregulation of SEPP1 and the downregulation of SELENBP1 in HCC patients.

## Figures and Tables

**Table 1:** Univariate and multivariate analysis of recurrence-free survival.

	Univariate Analysis				Multivariate Analysis			
	HR	Confidence Interval		*p*-Value	HR	Confidence Interval		*p*-Value
		Lower	Upper			Lower	Upper	
Sex (male)	2.46	1.24	4.86	0.010	2.33	1.14	4.75	0.02
Age (>60 years)	0.91	0.63	1.32	0.635				
Total Bilirubin (>20.4 μmol/L)	0.97	0.54	1.73	0.919				
Albumin (<35 g/L)	0.84	0.37	1.91	0.670				
Alanine aminotran-sferase (>50 U/L)	0.93	0.62	1.39	0.724				
γ-glutamyltransferas (>60 U/L)	1.85	1.27	2.68	0.001	1.39	0.91	2.13	0.126
Prothrombin time (>14 s)	0.94	0.49	1.80	0.855				
AFP (>400 ng/mL)	2.04	1.38	3.02	<0.001	1.69	1.09	2.60	0.018
HBsAg (+)	1.18	0.70	1.97	0.534				
Perihepatic invasion	3.50	1.29	9.52	0.014	4.09	1.40	11.92	0.010
PVTT	1.69	0.93	3.08	0.085	1.23	0.64	2.37	0.528
Tumor number (>2)	1.56	1.08	2.26	0.018	1.45	0.98	2.14	0.064
Maximum tumor diameter (>5 cm)	2.05	1.42	2.98	0.000	1.38	0.89	2.14	0.151
B cell (>166 cells/μL)	0.66	0.46	0.95	0.026	0.72	0.49	1.06	0.097
CD4^+^ T cell (>333 cells/μL)	1.61	0.75	3.46	0.221				
CD8^+^ T cell (>478 cells/μL)	0.65	0.42	0.99	0.046	0.62	0.40	0.96	0.031
CD4/CD8(>1.05)	1.35	0.74	2.46	0.321				
NK cell				0.010				0.004
lower (<120 cells/μL)	1.92	1.18	3.14	0.009	2.23	1.34	3.70	0.002
upper (>420 cells/μL)	1.64	1.09	2.48	0.018	1.57	1.03	2.41	0.038

HR, hazard ratio; AFP, α-fetoprotein; PVTT, portal vein tumor thrombus.

## Data Availability

The data used to support this research are included in this article, and the raw data are available from the National Genomics Data Center (OMIX001489).

## Supplementary Materials

The following supporting information can be downloaded at: https://www.mdpi.com/article/10.3390/biom14020222/s1, Supplementary Figure S1. The workflow of patient selection; Supplementary Figure S2. (A) The cells receiving 10× single-cell sequencing were divided into 32 clusters through dimensionality reduction clustering. (B) The expression of some marker genes in these cells; Supplementary Figure S3. Pathway signal enrichment analysis of the upregulated or downregulated genes in NK cells was conducted using metascape; Supplementary Tables S1–S4.
