# Supplementary material for: Peripheral Lymphocytes in Primary Liver Cancers: Elevated NK and CD8+ T Cells and Dysregulated Selenium Metabolism
Source: Biomolecules. 2024 Feb 14;14(2):222. doi: 10.3390/biom14020222 (PMC10886987; doi:10.3390/biom14020222)
Supplement: Supplementary file 1 [file biomolecules-14-00222-s001.zip › Supplementary figures.pdf]

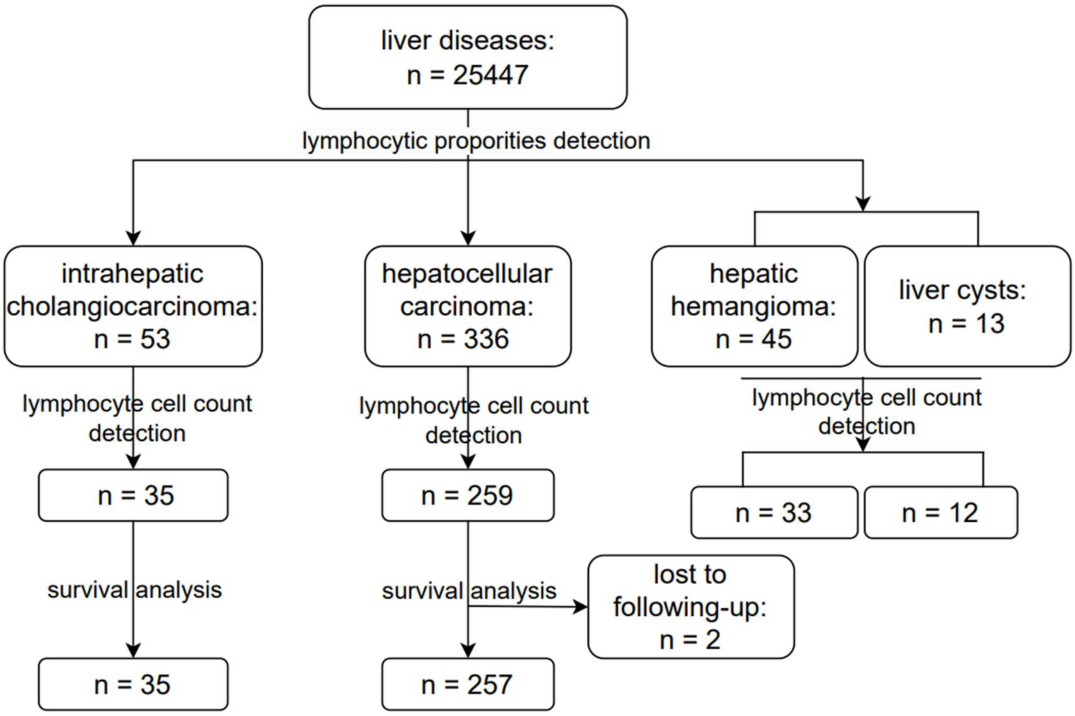

Supplementary Figure 1. The workflow of patient selection.

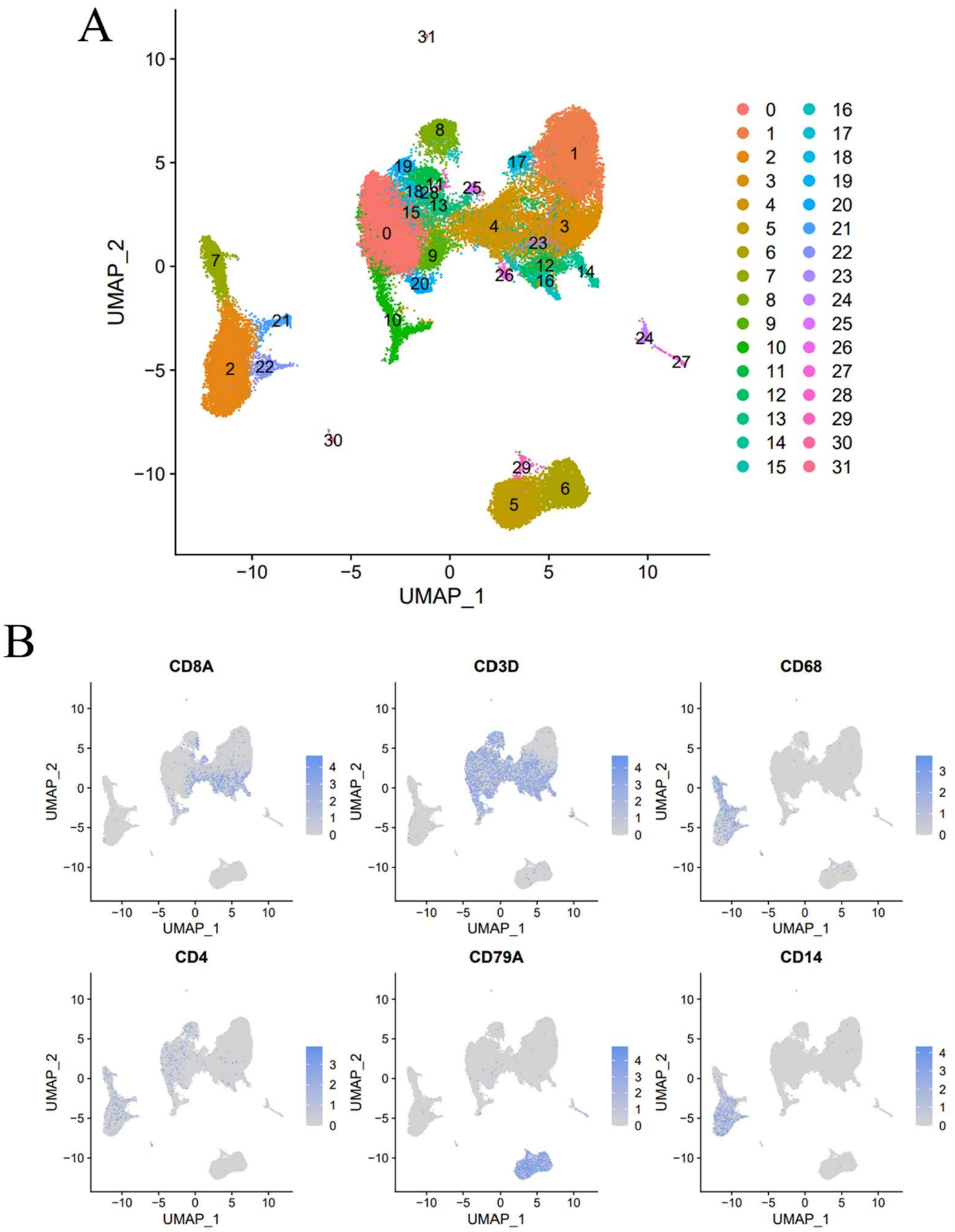

Supplementary Figure 2. (A) The cells receiving 10x single-cell sequencing were divided into 32 clusters through dimensionality reduction clustering. (B) The expression of some marker genes in these cells.

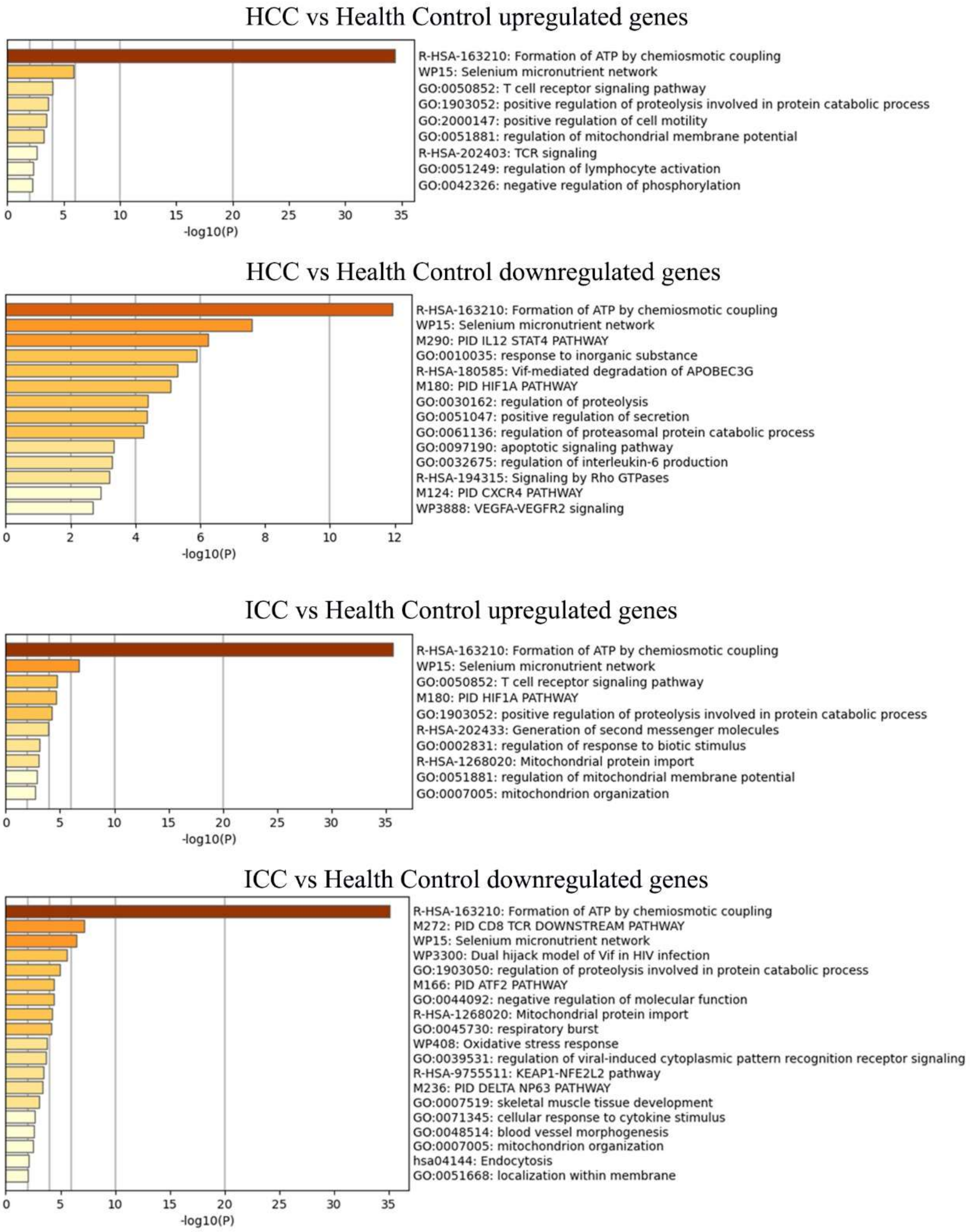

Supplementary Figure 3. Pathway signal enrichment analysis of the upregulated or downregulated genes in NK cells was conducted using *metascape*. The results showed that these differential genes were enriched in ATP formation and selenium metabolism pathways.

The impact of primary liver cancer on PBMCs manifested by alterations in cellular quantity, decreasing of TGF-beta signaling and dysregulated selenium metabolism.
